# Acceptability of a Self-Led Mindfulness-Based Intervention for Teens with Type 1 Diabetes: Pilot Randomized Controlled Trial

**DOI:** 10.2196/45659

**Published:** 2024-01-30

**Authors:** Tori Humiston, Caroline Cummings, Stephen Suss, Laura B Cohen, Holly Hazlett-Stevens, Amy Hughes Lansing

**Affiliations:** 1 Department of Psychological Sciences, University of Vermont Burlington, VT United States; 2 Department of Psychological Sciences, Texas Tech University Lubbock, TX United States; 3 Department of Psychology, Florida International University Miami, FL United States; 4 Department of Psychology, University of Nevada Reno, NV United States

**Keywords:** adolescents, diabetes distress, diabetes, health group intervention, intervention, mindfulness, psychosocial intervention, self-led mindfulness, type 1 diabetes

## Abstract

**Background:**

Diabetes distress among adolescents with type 1 diabetes has been associated with suboptimal diabetes outcomes, including lower quality of life, increased diabetes self-management challenges, and suboptimal glycemic outcomes.

**Objective:**

This study examined the feasibility and acceptability of a scalable self-led mindfulness-based intervention to reduce diabetes distress in adolescents with type 1 diabetes.

**Methods:**

Adolescents (N=25) aged between 14 and 18 years diagnosed with type 1 diabetes completed a baseline assessment. Participants were randomized to receive a 10-week self-guided mindfulness-based stress reduction workbook program (e-book or paper option) immediately (n=15) or after a 10-week wait (n=10). During the intervention period, participants completed weekly assignments and feedback surveys. At 10 weeks and 20 weeks, follow-up assessments were completed.

**Results:**

Findings indicated that participants did not find the original intervention feasible or acceptable. Adolescents reported barriers to completing the weekly material, such as that they forgot or that the material was not sufficiently related to their diabetes management. Adolescents also reported that a digital format rather than a workbook or e-book may be more acceptable. Results from weekly surveys provided the foundation for recommendations for future iterations of the mindfulness-based intervention for adolescents with type 1 diabetes.

**Conclusions:**

Participant feedback informed recommendations for self-led mindfulness programs for youth with type 1 diabetes. Adolescents indicated that a shorter, digital mindfulness-based intervention focused on diabetes-specific behaviors may be more helpful.

**Trial Registration:**

ClinicalTrials.gov NCT05115175; https://clinicaltrials.gov/study/NCT05115175

## Introduction

Adolescents with type 1 diabetes, an autoimmune disease that destroys insulin-producing cells in the pancreas, must engage in numerous daily health behaviors to manage their symptoms and prevent short- and long-term health complications [1]. The persistent challenges of disease management often result in diabetes distress, characterized as the burden, worries, and frustrations associated with diabetes [2]. Adolescents with greater diabetes distress experience lower quality of life, increased diabetes self-management challenges, and suboptimal glycemic outcomes (glycosylated hemoglobin; HbA_1c_) [3,4]. Heightened diabetes distress may affect type 1 diabetes outcomes both indirectly, through decreased engagement in disease self-management behaviors, and directly, through the effects of physiological arousal, hormone secretion, and insulin resistance on blood glucose levels and the microvascular system [5,6]. Current interventions aimed at fostering diabetes self-management in adolescence have targeted distress management [7] and quality of life [8], yet less research has examined mindfulness-based stress reduction (MBSR) interventions in the context of adolescents’ type 1 diabetes distress and self-management. This is despite cross-sectional data supporting the link between greater mindfulness, greater diabetes self-management, lower diabetes stress, and lower HbA_1c_ levels in adolescents with type 1 diabetes [9].

Increased mindfulness, enhanced through mindfulness-based interventions, is linked to numerous health benefits. One example is the MBSR intervention, a mind-body public health group intervention developed for medical patients to manage the stress of chronic medical conditions. MBSR consists of eight 2.5-hour-long, weekly sessions and 1 all-day practice session [10]. In adults with type 1 or type 2 diabetes, the benefits of mindfulness-based interventions include reduced distress and cardiovascular risk [11,12], and similar outcomes emerged for adolescents with chronic diseases generally [13]. More recently, mindfulness-based interventions have been considered in the context of improving type 1 diabetes management in emerging adults [14]. For example, among emerging adults with type 1 diabetes and suboptimal glycemic levels, an MBSR intervention was found to improve psychosocial outcomes but not glycemic outcomes and was highly acceptable [15,16]. Further, an additional study within the type 1 diabetes context found that a brief self-compassion intervention, which is a core component of MBSR, was acceptable and feasible among adolescents with disordered eating and improved mindfulness and coping [17]. These findings support the possible benefits of improved mindfulness in reducing diabetes distress and improving diabetes self-management in adolescents with type 1 diabetes.

The goal of this study was to begin an iterative approach to developing a self-led, scalable MBSR intervention for adolescents with type 1 diabetes. The intervention was modeled on a 10-week bibliotherapy MBSR program implemented by Hazlett-Stevens and Oren [18] that was found to be feasible and acceptable for college students and to reduce distress and anxiety for those who completed the program. A self-led MBSR program approach may be particularly helpful for disseminating MBSR to adolescents with type 1 diabetes, a population that already experiences the intensive time burden of diabetes management [19]. This study examined the acceptability, feasibility, and potential utility of a 10-week self-led workbook (e-book or paper option) MBSR intervention for adolescents with type 1 diabetes through a randomized waitlist control design. It was hypothesized that participation in a self-led MBSR intervention would be feasible and acceptable, as evidenced by low treatment attrition and positive participant feedback. Finally, we provide recommendations for future iterations of self-led, digital MBSR interventions for adolescents with type 1 diabetes based on recommendations by participants.

## Methods

### Ethical Considerations

All procedures and documents were approved by the institutional review board at the University of Nevada, Reno (1221205). Web-based parental consent was obtained for children aged 18 years or younger, in which case child assent was also obtained. Participants 18 years of age or older provided web-based consent. Participants earned US $10 for each assessment (up to US $30), US $10 for completing at least 6 of the 10 weekly surveys over the 10-week intervention period, and an additional US $10 for completing all 10 weekly surveys. The maximum earnings for each participant were US $50, in the form of an Amazon gift card. All measures were completed electronically through Research Electronic Data Capture (REDCap; Vanderbilt University). Participants were informed during the informed consent process that their answers and data would not be shared with individuals outside of the research team unless the research team was concerned about the participant’s safety.

### Participants

Participants for this study included a sample of 25 adolescents (n=14, 56% female) aged between 14 and 18 years (mean age 16.25, SD 1.6 years) from urban and rural areas of Nevada. Most participants identified as White (n=22, 88%), 1 participant as Asian, 1 participant as Native Hawaiian or Pacific Islander, and 1 participant as biracial. Participants reported a wide range of diagnosis lengths, from less than a year to 16.15 years (mean diagnosis length 5.3, SD 4.1 years). A total of 19 (76%) participants reported using a continuous blood glucose monitor, and 17 (68%) endorsed using an insulin pump. Additionally, through self-report, 10 (40%) participants reported that they qualify for free lunch at school. Inclusion criteria included being an adolescent (aged between 13 and 19 years) with a type 1 diabetes diagnosis currently attending school or being a recent high school graduate. Participants were excluded if they were wards of the state, had severe psychiatric disturbances (eg, active psychosis), or had severe developmental delays that hindered their ability to self-report. Participants were not excluded based on the length of their type 1 diabetes diagnosis.

### Procedures

Participants were recruited with flyers at a regional diabetes camp, flyers sent to community diabetes support groups, and direct recruitment by research staff in a local pediatric endocrinology clinic. All enrolled participants completed a baseline survey, including a self-report of their most recent HbA_1c_ percentage value, and were randomized to either begin the 10-week intervention period immediately or after 10 weeks, with participants randomized to start the intervention after the 10-week waiting period acting as a control group. Randomization was computerized and stratified based on sex, duration of type 1 diabetes diagnosis (2 years or less vs more than 2 years), and most recent HbA_1c_ (8.5% or below vs 8.6% or above). Participants also completed assessment questionnaires 10 weeks and 20 weeks after the study start date and a weekly survey for the 10 weeks of intervention participation.

**Figure 1:**
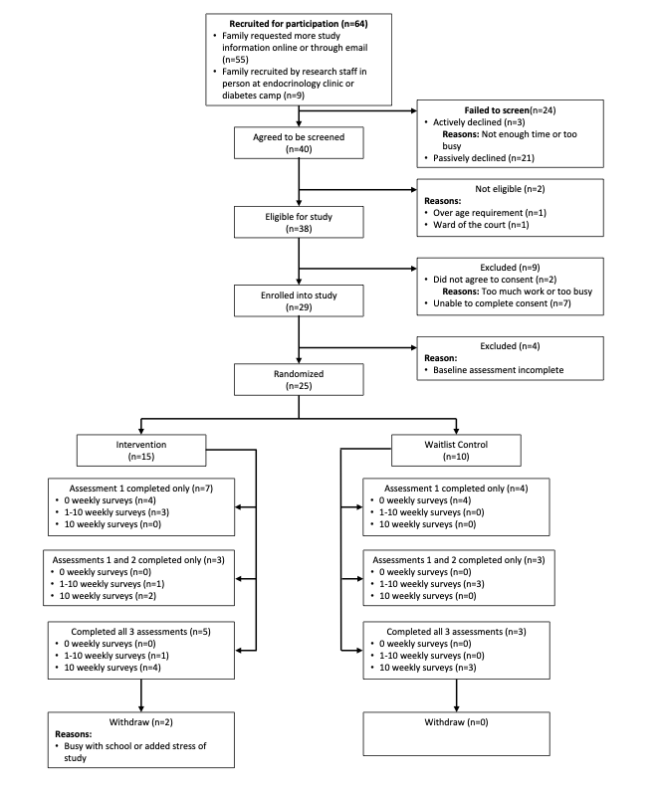
CONSORT (Consolidated Standards of Reporting Trials) table of participation.

Recruitment for this project began in the fall of 2018 and was completed in early spring 2020. Research staff were trained by 2 graduate students on recruitment procedures and eligibility criteria. A total of 64 participants contacted our research staff, indicating interest in participating in the study and providing permission to contact them either on the internet or in-person (Figure 1). Of the 64 participants who indicated interest in the study, 5 declined further screening, and 2 were not eligible after screening. During the study period, after 29 participants had consented and enrolled, it was determined, due to feedback from those enrolled participants as well as attrition and loss of follow-up, that the intervention required revision to increase acceptability; accordingly, recruitment was stopped at that time. An additional 28 participants expressed interest in enrolling but did not complete the consent process before the interim study end point was reached; this was also before the original goal of 60 participants was reached. Participant recruitment and enrollment are further discussed below as part of the analysis of feasibility and acceptability.

### Intervention Program

The mindfulness-based intervention in this study was delivered through a teen MBSR workbook, either an e-book or paper workbook, and web-based communication across a 10-week period. If selected, the paperback workbook was mailed to participants’ home address; otherwise, access to the e-workbook was provided through email. Participants were assigned weekly readings and activities from an MBSR workbook [19]. Topics included understanding stress, an introduction to mindfulness, and mindfulness-based exercises recommended to be completed daily (eg, mindful eating). Mindfulness-based exercises were either self-led per instructions provided in the workbook or completed using audio-recorded instructions. Participants received emails twice per week with reminders about the current week’s assignments and to practice mindfulness each day. At the end of the week, participants received a reminder to complete a brief survey on the acceptability of that week’s content. This survey included both multiple-choice questions and open-ended questions regarding how helpful participants found each exercise and what hindered them from completing the exercises that week. Participants completed the following measures at baseline and immediately following intervention completion.

### Measures

Multiple measures were administered in the study. Only those relevant to this study are described below.

#### HbA1c Percentage

At intake, participants self-reported their most recent HbA_1c_ level. Higher HbA_1c_ percentages are associated with less optimal glycemic control in the past 2-3 months.

#### Intervention Acceptability and Feedback for Recommendations

Acceptability was measured both objectively based on participant engagement as well as per participant report. First, attrition was used as a proxy variable for acceptability. Second, at the end of each week of the intervention, participants completed both multiple-choice and open-ended questions pertaining to the intervention content that week. Information from weekly surveys was used to create recommendations for future intervention development. Specifically, the following questions were asked: “How much of the suggested readings from the book did you read over the past week?” (0=“not at all” to 5=“I read all of the suggested readings”) “What percentage of exercises from the suggested readings for the previous week did you complete while reading the chapter?” (0=“not at all” to 5=“I read all of the suggested exercises”) “How often did you engage in the exercises suggested in the chapter during the previous week?” (0=“none” to 3=“almost daily”) “What got in the way of you following the workbook?” (0=“I forgot”; 1=“I didn’t have time”; 2=“I didn’t understand the material,” “the material was too difficult to follow,” or “the instructions were unclear”; or 3=“other: explain”) and “Please tell us any other thoughts you had on the workbook readings, weekly exercises, or audio tracks this week” (open-ended).

### Analyses

To assess the feasibility and acceptability of the self-led MBSR intervention for adolescents with type 1 diabetes, we conducted descriptive statistics of level of attrition and quantitative questions from participants’ weekly feedback surveys. Qualitative feedback from the weekly feedback surveys was brief and informally and visually analyzed and summarized. We created recommendations for a more acceptable mindfulness-based intervention based on the feedback from the weekly surveys.

## Results

All feasibility and acceptability analyses pointed to issues with the acceptability and feasibility of the initial intervention model. First, consistent with problems with study feasibility, many participants did not complete the full study period and were lost to follow up. Among the 29 enrolled participants, 25 completed their baseline survey and were randomized to intervention now (n=15) or waitlist (n=10); groups were not balanced as enrollment was stopped early. A total of 4 of the 29 participants passively declined to complete the baseline survey and were not randomized. Overall, 2 participants withdrew from the study after intake (reasons: busy with school and study participation was stressful). Additionally, 2 participants were lost to follow-up during the program, and 7 participants were lost to follow-up at 10 weeks (3 from intervention-first and 4 from the waitlist group). Another 6 were lost to follow-up at 20 weeks, 3 of those from the intervention first group that was now completing extended follow-up. This suggested challenges with the waitlist design at a minimum, as well as with maintaining participant engagement in the study process.

Second, we examined participant feedback on both reasons for attrition and low engagement. The indicated reasons for attrition included the conflict of busy schedules and the weekly time commitment for the intervention. In the weekly surveys, participants reported, through a Likert scale, barriers to engaging in the weekly material. Options for barriers were “I forgot,” “I didn’t have time,” “I didn’t understand the material or instructions,” and “other.” Average frequencies of selected barriers indicated that the most common barrier to engaging in the material was lack of time (50/120, 41.7%). Average frequencies for the 3 other barriers included 21.7% (26/120; “I forgot”), 23.3% (28/120; “other”), and 13.3% (16/120; “I didn’t understand the material”). When participants selected “other,” they were prompted to fill in a textbox describing the barrier. For the participants who did enter a reason in the textbox, responses included that they felt they did not need a mindfulness practice and noted that diabetes-specific activities would be more helpful than general activities. Additional responses included that the workbook intervention format reflected school tasks and that some of the reading and activities felt geared toward a younger audience. Textbox responses were brief, categorized by 2 graduate students, and reviewed by the principal investigator. However, due to the brevity of responses, no formal codebook was used to code responses. Feedback on acceptability and feasibility was integrated, and recommendations for a digitally tailored MBSR intervention for teens with type 1 diabetes were generated by the study team, including experts in adolescent diabetes management and mindfulness-based interventions.

## Discussion

### Overview

This study evaluated the feasibility and acceptability of a self-led MBSR intervention. Although there was no support for the feasibility and acceptability of the intervention model, participants provided feedback on multiple improvements that would enhance the feasibility and acceptability of the intervention program. Participants’ weekly feedback pointed to multiple domains where an MBSR intervention might be tailored to the type 1 diabetes context. Hurdles remain in understanding the feasibility and acceptability of these programs, especially in more scalable, self-led forms. For example, in the type 1 diabetes context, there are limited studies examining the feasibility of mindfulness-based interventions for young persons of color with type 1 diabetes populations [15]. Additional research on mindfulness-based interventions for adolescents with type 1 diabetes indicates that brief, digital sessions may be more acceptable and feasible for this population [17]. Further research is needed to clarify the long-term benefits and acceptability of mindfulness-based interventions on diabetes distress and glycemic outcomes for adolescents with type 1 diabetes.

Our research has identified multiple potential areas for increasing the acceptability of a self-led mindfulness program for adolescents with type 1 diabetes. In this intervention, feasibility and acceptability assessments indicated that adolescents prefer a shorter, web-based intervention focused on mindfulness regarding diabetes-specific behaviors. We have since identified 5 potential avenues for future mindfulness intervention development in the adolescent type 1 diabetes context that would address the concerns raised by the adolescents in our sample while maintaining a connection to mindfulness-based therapy approaches. First, decrease the amount of content in the self-led form to emphasize the connections between everyday mindfulness, acceptance skills, and diabetes management. Some participants indicated that the original modules and instructions were difficult to understand. Therefore, it may be beneficial to focus the program on a few basic mindfulness skills that are connected to diabetes management to help build an insightful mindfulness repertoire that is meaningful to daily diabetes management. For example, the early sessions of a self-led mindfulness-based intervention might teach an introduction to mindfulness with a guided body scan aimed at increasing self-compassion around diabetes symptoms and tasks. Body scans are a foundational mindfulness practice in MBSR and can be modified to emphasize self-compassion for physiological sensations and experiences that occur during diabetes management (wearing diabetes technology or experiencing hyper- or hypoglycemia). Following this early session, adolescents might be guided to engage in a formal practice audio recording of a diabetes-oriented body scan. Follow-on sessions might continue this same training and expand the experience with a diabetes-oriented body scan with new attention toward self-monitoring and glycemic awareness (symptoms of hypo- or hyperglycemia). Increasing interoceptive awareness through guided meditation may foster adolescent awareness of hyper- and hypoglycemia. Further, acceptability and feasibility may increase if the intervention were delivered through a website or mobile app. Second, the acceptability of a self-led mindfulness-based intervention for adolescents with type 1 diabetes might be increased by contextualizing the emotionally evocative nature of diabetes-management behaviors (changes in eating patterns and emotions in social environments) as life events where mindfulness may be a useful practice. For example, additional sessions might teach mindful eating practices to increase nonjudgmental awareness of food cravings or other hunger- or thirst-related diabetes experiences. Building mindful eating practices may also increase self-compassion about the changes in eating habits necessary to maintain on-target glycemic levels. In addition, mindful eating practice may also help build a repertoire of acceptance and nonjudgmental awareness to decrease diabetes distress when eating habits and glycemic levels are not aligned with medical professionals’ guidelines for type 1 diabetes management. Third, It may also be beneficial for acceptability to incorporate dialectical behavior skills into the mindfulness practice to balance the notion that diabetes-management behaviors are not naturally reinforcing (eg, finger sticks and pump site or sensor placements), yet they are still necessary to maintain health. Later sessions might introduce and teach mindful stopping for stress management to teach adolescents how to pause and engage in mindful savoring of positive life events when diabetes is challenging. Incorporating mindful stopping skills for diabetes-related stressors and activities in everyday life, as well as rumination about past and future diabetes challenges, might particularly benefit reductions in diabetes distress. Fourth, the acceptability of self-led mindfulness programs might be increased by adding a peer support component or a parent component. Although Ellis and colleagues [16] did not find links between a mindfulness-based intervention and improved glycemic outcomes, they did find that a control group peer support program did effectively improve glycemic outcomes, despite the peer support aspect of the program not focusing on diabetes management. The group learning environment is a key feature of live MBSR training that is not available in a self-led format. Periodic (eg, quarterly or monthly) web-based chat rooms or discussion boards moderated by a support staff member might allow a space to encourage peer support around learning self-led mindfulness skills without requiring the greater costs and time invested in live group-based MBSR programs. Further, adding a parent component may help reinforce skills that are learned and support a positive parent-adolescent relationship, the latter of which has downstream effects on glycemic control in this pediatric population [20,21]. Finally, future studies may benefit from the following methodological changes. First, researchers may consider incorporating the use of focus groups and semistructured interviews, as well as other approaches toward conducting community-based participatory research, to obtain information on intervention content and acceptability before recruitment. Second, researchers may consider monitoring recruitment for a representative sample and incorporate recommendations for race-conscious research [22,23] when examining if mindfulness is appropriate in light of historical antecedents.

These recommendations would benefit from testing and exploration in future research studying self-led mindfulness-based interventions in adolescents with type 1 diabetes.

### Limitations

This study is limited by multiple factors. The sample that consented to the study was predominantly White and therefore is not generalizable to other races and ethnicities. The study was also limited in the scope of the time period in which participants were recruited and completed the study. Some participants had already completed the study before the COVID-19 pandemic, while others completed the study during the pandemic, which may have confounded the stress and mindfulness levels of the participants. Due to the sample size, we were not able to analyze the data for potential differences across participants who were enrolled before and during the COVID-19 pandemic. Most of the participants reported an HbA_1c_ that was 8.5% or lower, which may limit the generalizability of the recommendations to adolescents with a higher HbA_1c_. Although not asked in this study, it may be helpful for future studies to ask participants about their previous history with mindfulness and meditation.

### Conclusions and Future Directions

While keeping the limitations in mind, the findings of this study provide important data to contextualize the content and delivery of mindfulness-based interventions for adolescents with type 1 diabetes and provide guidance for developing an acceptable and scalable self-led mindfulness-based intervention. First, mindfulness and stress-related processes may be particularly important to understand in the context of type 1 diabetes management, given the long-term health complications that involve the vascular system. For example, the primary cause of death in middle-aged and older adults with type 1 diabetes is cardiovascular disease [24], and cardiovascular disease as well as other macro- and microvascular complications [25] contribute to an almost 17-year decrease in life span for those with an early age of type 1 diabetes onset [1]. Mindfulness-based interventions have been shown to also diminish chronic biological stress regulatory system activation that directly contributes to cardiovascular disease [26], highlighting the importance of developing mindfulness-based interventions that are acceptable and feasible for adolescents and emerging adults with type 1 diabetes. Further research is needed to examine the long-term physiological effects associated with mindfulness-based interventions for individuals with type 1 diabetes and the potential cardio-protective benefits.

Second, the data from study participants provided critical information for guiding the development of scalable self-led mindfulness-based intervention models, including emphasizing content that directly links mindfulness practice to diabetes management with the inclusion of diabetes-specific mindfulness activities. Further research examining the acceptability and effects of self-led mindfulness-based interventions that meet the recommendations provided herein and within a sample of youths that includes persons of color and with socioeconomic disadvantages is needed to better understand and support research on mindfulness interventions and type 1 diabetes management in adolescents.

Finally, mindfulness-based interventions might have a role in addressing internalizing psychopathology that is known to impact glycemic outcomes and quality of life for adolescents with type 1 diabetes. Depressive symptoms may contribute to an environment where adolescents experience decreased energy, leading to decreased engagement in diabetes-management behaviors. Decreased energy then contributes to a coercive cycle where decreased glycemic control contributes to greater depressive symptoms [27]. Adolescents may also experience anxiety symptoms, such as worry. Rechenberg and colleagues [28] found that adolescents with type 1 diabetes reported increased worry about managing their health, particularly regarding hypoglycemia and correct insulin dosing. Mindfulness-based interventions may be useful in disrupting this cycle by reducing the underlying stress associated with many psychological concerns. Further research is needed to identify if subpopulations of adolescents with type 1 diabetes, such as those with internalizing symptoms, might particularly benefit from self-led mindfulness-based interventions.

## Abbreviations

HbA1c: glycosylated hemoglobin
MBSR: mindfulness-based stress reduction

## Data Availability

The data sets generated and analyzed during this study are not publicly available as participants were informed during the informed consent process that their answers and data would not be shared with individuals outside of the research team unless the research team was concerned about the participant’s safety.
